# Soil degradation and recovery – Changes in organic matter fractions and structural stability

**DOI:** 10.1016/j.geoderma.2020.114181

**Published:** 2020-04-01

**Authors:** Johannes L. Jensen, Per Schjønning, Christopher W. Watts, Bent T. Christensen, Peter B. Obour, Lars J. Munkholm

**Affiliations:** a Department of Agroecology, Aarhus University, Blichers Allé 20, 8830 Tjele, Denmark; b Department of Sustainable Agricultural Sciences, Rothamsted Research, Harpenden, Hertfordshire AL5 2JQ, United Kingdom; c Department of Natural Resources and Environmental Sciences, University of Illinois at Urbana Champaign, 1102 S. Goodwin Ave., MC-047, Urbana, IL 61801, USA

**Keywords:** A, Arable, AG, Arable converted to grass, BF, Bare fallow, CEC, Cation exchange capacity, DI, Clay-SOM disintegration, DispClay 1–2 mm, Clay dispersibility of 1–2 mm aggregates, DispClay 8–16 mm, Clay dispersibility of 8–16 mm aggregates, *E*, Young’s modulus, *E*_sp_, Mass-specific rupture energy, G, Grass, GA, Grass converted to arable, GBF, Grass converted to bare fallow, HWC, Hot water-extractable carbon, POXC, Permanganate oxidizable carbon, SSA, Specific surface area, SSS, Soil structural stability, *Y*, Tensile strength, Soil restoration, Soil degradation, Rate of change, Soil structural stability, Soil organic carbon, Soil management

## Abstract

The combination of concurrent soil degradation and restoration scenarios in a long-term experiment with contrasting treatments under steady-state conditions, similar soil texture and climate make the Highfield land-use change experiment at Rothamsted Research unique. We used soil from this experiment to quantify rates of change in organic matter (OM) fractions and soil structural stability (SSS) six years after the management changed. Soil degradation included the conversion of grassland to arable and bare fallow management, while soil restoration comprised introduction of grassland in arable and bare fallow soil. Soils were tested for clay dispersibility measured on two macro-aggregate sizes (DispClay 1–2 mm and DispClay 8–16 mm) and clay-SOM disintegration (DI, the ratio between clay particles retrieved without and with SOM removal). The SSS tests were related to soil organic carbon (SOC), permanganate oxidizable C (POXC) and hot water-extractable C (HWC). The decrease in SOC after termination of grassland was greater than the increase in SOC when introducing grassland. In contrast, it was faster to restore degraded soil than to degrade grassland soil with respect to SSS at macro-aggregate scale. The effect of management changes was more pronounced for 8–16 mm than 1–2 mm aggregates indicating a larger sensitivity towards tillage-induced breakdown of binding agents in larger aggregates. At microscale, SSS depended on SOC content regardless of management. Soil management affected macroscale structural stability beyond what is revealed from measuring changes in OM fractions, underlining the need to include both bonding and binding mechanisms in the interpretation of changes in SSS induced by management.

## Highlights

• The rate of change in soil structural stability and SOM fractions were quantified.
• SOC was mainly affected by C input and tillage.
• It was faster to lose than to gain SOC.
• At macroscale, it was faster to gain than to lose soil structural stability.
• SOM fractions were not able to explain the dynamics at macroscale.

## 1 Introduction

Soil aggregation and soil structural stability (SSS) play a significant role in soil organic C (SOC) sequestration as stable aggregates protect soil organic matter (SOM) against decomposition (Six et al., 2004). Further, SSS links to loss of particle-associated pollutants (de Jonge et al., 2004), soil erosion (Le Bissonnais, 1996), soil cementation and seedbed quality (Kay and Munkholm, 2004).

The quantity and quality of SOM are main drivers in the formation and stabilization of soil structure in most soils with different SOM bonding and binding agents being important at different soil structural levels (Abiven et al., 2009, Bronick and Lal, 2005, Tisdall and Oades, 1982). Bonding relates to gluing mineral particles together by decomposition products (e.g. polysaccharides), while binding refers to enmeshment of aggregates by plant roots and fungal hyphae (Tisdall and Oades, 1982). At micro-aggregate level (<250 µm), flocculation of clay and SOM, cementation of dispersed clay, and bonding agents from plants, soil fauna and microbes add to SSS (Chenu, 1989, Haynes and Swift, 1990). At macro-aggregate level (>250 µm), cross-linking and enmeshment by fungal hyphae and plant roots are crucial for SSS (Miller and Jastrow, 1990). Micro-aggregates are more stable than macro-aggregates, and less affected by management and SOM, while stabilization of macro-aggregates is controlled mainly by management and SOM levels (Oades, 1984).

Permanganate oxidizable C (POXC) and hot water-extractable C (HWC) have been considered as labile SOM fractions more sensitive to management than total SOC (Culman et al., 2012, Ghani et al., 2003). Bongiorno et al. (2019) found that POXC can provide information about soil physical condition, and suggested POXC as a comprehensive soil quality indicator, while Fine et al. (2017) claimed POXC to be the best single predictor for soil health.

Only a few studies have related changes in land use to changes in SOM fractions and SSS (e.g., Perfect et al., 1990). The quantification of rates of change in SSS and knowledge of links between SSS and SOM fractions is beneficial for restoring degraded soil and identifying sustainable management of soils with adequate SSS. One outstanding issue is whether degradation and restoration occurs at a similar rate in relation to both SOM fractions and SSS.

The objective of this study was to quantify the effects of different SOM fractions on SSS in soil subjected to degradation and restoration managements. Permanent grassland was used as reference treatment. Changes in SOM content due to management affect SSS differently at different spatial scales. At <20 µm scale, extremely stable SOM-mineral interactions are responsible for SSS. Accordingly, we applied an extreme clay-SOM disintegration test to reveal differences at microscale. We hypothesize that SSS at microscale change more slowly than SOM content in both degradation and restoration managements. Further, we applied a clay dispersibility test with low degree of disturbance to 1–2 mm and 8–16 mm rewetted macro-aggregates to investigate if the rate of change in SSS was scale-dependent. We hypothesize that SSS at macroscale changes more rapidly than SOM contents in soil under both degradation and restoration managements.

Soils were from the Highfield land-use change experiment at Rothamsted Research (Highfield-LUCE), sampled six years after changes in managements. This ensured that soil degradation and restoration management were initiated simultaneously on a site with a well-known history, with long-term treatments under steady-state conditions, and without confounding effects of differences in soil type, soil texture and climate. The changes in management were profound making this experiment ideal for investigating shorter-term effects on SOM fractions and SSS.

## 2 Materials and methods

### 2.1 The Highfield land-use change experiment and treatments

The Highfield ley-arable experiment at Rothamsted Research, Harpenden, UK (51°80′N, 00°36′W) was initiated in 1949 (Johnston, 1972). Its purpose was to look at the effects of different cropping systems on yield and SOM. Highfield had been in permanent grass since 1838; on this site some plots stayed in permanent grass, others went into continuous arable cropping and some alternated between leys and arable. It has taken about 60 years for soils to reach a steady-state condition following changes in the management systems (Hirsch et al., 2017, Rothamsted Research, 2018).

**Fig. 1:**
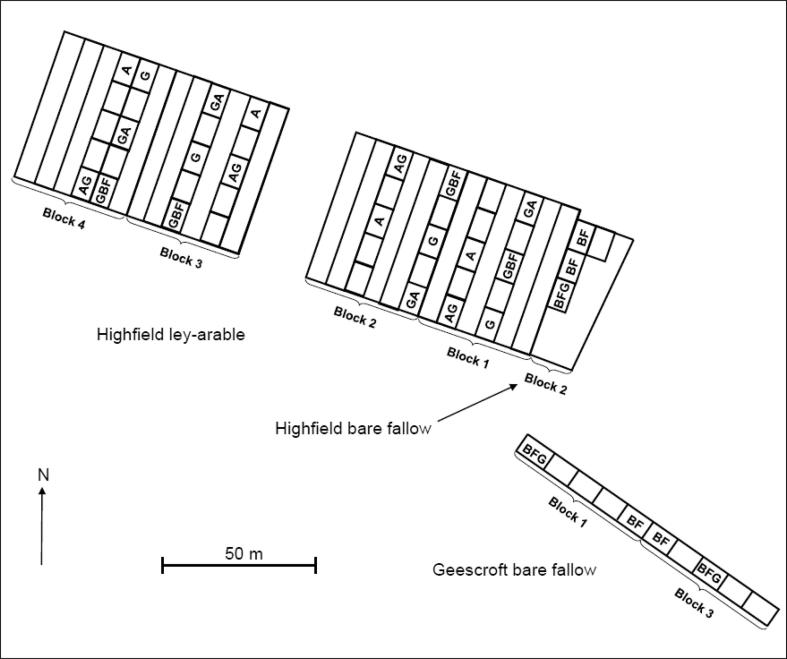
Distribution of plots in Highfield showing the arable (A), arable converted to grass (AG), grass converted to bare fallow (GBF), grass converted to arable (GA) and grass (G) treatments in blocks 1–4 of the ley-arable experiment, and the bare fallow (BF) and bare fallow converted to grass (BFG) treatments in blocks 1–3 of the adjacent bare fallow experiments.

In 2008, 10 × 6 m areas within the existing arable (A) and grass (G) plots on the Highfield ley-arable site were converted to bare fallow, arable or grass, while other areas remained unchanged. Likewise, in 2008, 10 × 6 m areas within the existing bare fallow (BF) plots on the Highfield bare fallow and Geescroft bare fallow sites (located adjacent to the Highfield ley-arable experiment) were converted to arable or grass. The long-term BF treatment was established in 1959. For this study, we selected three conversion treatments in the ley-arable experiment: Arable converted to grass (AG), grass converted to bare fallow (GBF) and grass converted to arable (GA). We also selected the conversion of bare fallow to grass (BFG) in the Highfield bare fallow and Geescroft bare fallow sites (Fig. 1).

The AG treatment is sown with a mixture of meadow fescue (*Festuca pratensis* L.), timothy-grass (*Phleum pratense* L.) and white clover (*Trifolium repens* L.). The grass/clover ley receives no N fertilizer and the biomass is cut and removed in early summer. The small amount of regrowth is topped in early autumn and left on the plots. The GBF treatment is plowed or rotavated two to four times a year to keep any plant regrowth to a minimum. The GA treatment was sown with winter cereals (winter wheat, *Triticum aestivum* L. and winter oats, *Avena sativa* L. in rotation). The winter cereals are fertilized with 220 kg N ha^−1^ y^−1^ and straw is removed. The conversion to grass in BFG was as described for AG. The plowing depth in A, GBF, GA and BF was 23 cm. The A, AG, G, GA and BFG plots were fertilized with 65 kg P ha^−1^ and 250 kg K ha^−1^ every three years.

The A, AG, G, GA and GBF treatments were part of a randomized block design with four field replicates, whereas the four BF and three BFG plots were located adjacent to the experiment (Fig. 1). The soil is a silt loam soil belonging to the Batcombe series and is classified as an Aquic Paludalf (USDA Soil Taxonomy System) and Chromic Luvisol (WRB) (Watts and Dexter, 1997). For a more detailed description of the long-term treatments, see Jensen et al. (2019). Basic soil characteristics for BF, A and G treatments have been reported previously along with SOC, POXC, HWC, clay dispersibility of 1–2 mm aggregates and clay-SOM disintegration (Jensen et al., 2019). Hirsch et al., 2017, Todman et al., 2018 focused on biological aspects in the Highfield-LUCE.

### 2.2 Soil sampling

Soil was sampled in March 2015 six years after the initiation of the Highfield-LUCE. Sampling was done at field capacity corresponding approximately to a soil water potential of −100 hPa. Soil blocks (~2.75 l) were carefully retrieved from the 6–15-cm soil layer by use of a spade. Three soil blocks were sampled from randomly chosen sites within each experimental plot. The soil was kept in sturdy containers to prevent soil disturbance during transport and stored in a field-moist condition at 2 °C until required for analysis. Soil from the blocks was spread out in steel trays at room temperature, carefully fragmented by hand in several sittings along natural planes of weakness, and finally left to air-dry.

### 2.3 Basic chemical and physical analysis

The texture of air-dried bulk soil (crushed and passed through a 2-mm sieve) was determined by the hydrometer method for clay (<2 μm) and silt (2–20 μm) content and the sieve method for mineral particles > 63 μm (Gee and Or, 2002). The soil was tested for carbonates by adding a few droplets of 10% HCl, but none was found. Soil organic matter was removed with H_2_O_2_ before estimation of clay and silt as described in Jensen et al. (2017). The SOC content was determined on ball-milled subsamples using high-temperature dry combustion (Thermo Flash 2000 NC Soil Analyzer, Thermo Fisher Scientific, Waltham Massachusetts, USA). Specific surface area (SSA) was determined by the ethylene glycol monoethyl ether method (Petersen et al., 1996), and cation exchange capacity (CEC) was determined after Kalra and Maynard (1991). Soil pH was determined in 0.01 M calcium chloride (CaCl_2_) solution (1:2.5, *w*/w). The properties were determined at plot level.

### 2.4 Soil organic matter fractions

Permanganate oxidizable carbon (POXC) was determined at plot level on air-dry 2-mm sieved soil following Culman et al. (2012) and as detailed in Jensen et al. (2019). In short, soil was shaken in a potassium permanganate (KMnO_4_) solution and allowed to settle after which the supernatant was transferred, absorbance measured and finally converted to a POXC quantity.

Hot water-extractable carbon (HWC) was determined at plot level on air-dry 2-mm sieved soil following Ghani et al. (2003) and as detailed in Jensen et al. (2019). Briefly, soil was shaken in water at 20 °C, centrifuged, and the supernatant decanted. The soil was re-suspended in water, shaken for 16 h at 200 rpm and 80 °C, centrifuged, and the supernatant was filtered after which HWC was determined.

### 2.5 Soil structural stability and strength

Clay dispersibility was determined at plot level on 1–2 mm aggregates (DispClay 1–2 mm) isolated from the air-dry 2-mm sieved soil, and on 8–16 mm aggregates (DispClay 8–16 mm) isolated by sieving the air-dry bulk soil. The method is described in detail in Jensen et al. (2019). In short, the aggregates were adjusted to a matric water potential of −100 hPa on tension tables, shaken in artificial rainwater (0.012 mM CaCl_2_, 0.150 mM MgCl_2_, and 0.121 mM NaCl; pH 7.82; EC 2.24 × 10^−3^ S m^−1^), and the suspension left to stand after which ≤2 µm particles was siphoned off. The weight of dispersed clay was determined after oven-drying (105 °C for 24 h). The sediment was corrected for particles > 250 µm for DispClay 1–2 mm and for particles > 2 mm for DispClay 8–16 mm, both isolated by chemical dispersion. This was done to relate clay dispersibility to soil free of particles > 250 µm and stone-free soil for DispClay 1–2 mm and DispClay 8–16 mm, respectively.

Clay-SOM disintegration (DI) and soil aggregate strength were estimated on bulked soil for each plot as outlined as follows. Particles ≤ 2 µm estimated with no H_2_O_2_-removal of SOM prior to soil dispersion was measured as described by Jensen et al. (2017), and DI was calculated as the ratio between clay particles retrieved without and with SOM removal. Soil with DI values < 1 kg kg^−1^ can be regarded as being extremely stable since they have resisted disintegration after end-over-end shaking for 18 h in sodium pyrophosphate.

Aggregate strength was determined on 8–16 mm aggregates isolated from the air-dry bulk soil as detailed in Obour et al. (2018). Briefly, tensile strength (*Y*) was tested for 15 randomly selected aggregates per plot by subjecting them to an indirect tension test comprising crushing between two parallel plates. The point of failure for each aggregate was automatically detected when a continuous crack or sudden drop in force (40% of the maximum load) was read. After the test, the crushed aggregates were oven-dried at 105 °C for 24 h to determine their gravimetric water content. The calculation of tensile strength (*Y*), mass-specific rupture energy (*E*_sp_) and Young’s modulus (*E*) was as described in Obour et al. (2018) except for the calculation of the effective diameter used in the calculation of *Y*, where the mean dry mass of all aggregates instead of the mean dry mass of aggregates at plot level was used. Further, *E* was determined by manually selecting two points on the stress–strain curve for each aggregate.

### 2.6 Calculations and statistics

The soil properties measured in this study are expressed as an oven-dry weight mass proportion (105 °C for 24 h) of the mineral fraction. The properties include particle size fractions, SOC, POXC, HWC, SSA, CEC, DispClay 1–2 mm and DispClay 8–16 mm.

For the statistical analysis, the R-project software package Version 3.4.0 (R Foundation for Statistical Computing) was used. Treatment effects were analyzed with a linear mixed model including block as a random effect when comparing A, AG, G, GA and GBF. The criterion used for statistical significance of treatment effects was *P* < 0.05. When the treatment effect was significant, further analyses were made to isolate differences between treatments (pairwise comparisons) using the general linear hypotheses (*glht*) function implemented in the R *multcomp* package and the Kenward-Roger method to calculate degrees of freedom (Kenward and Roger, 2009). Treatment effects for the comparison of BF and BFG were analyzed separately since the BF and BFG treatments were located at one end of the experiment in its own design (Fig. 1). Treatment differences for the comparison of the BF and BFG treatments and the GBF and G treatments were based on a pair-wise *t*-test, acknowledging that this is a less robust test, and that the treatment differences could be due to soil variation since the BF and BFG treatments are not a part of the original ley-arable experiment. Logarithmic (ln) transformation was performed on *Y*, *E*_sp_ and *E* to yield normality. For models with more than one predictor, the adjusted coefficient of determination (R^2^) is reported. Akaike’s information criterion (AIC) was used to compare models with different numbers of parameters (Akaike, 1973).

## 3 Results

### 3.1 Basic soil characteristics

Generally, contents of clay, silt and sand did not differ significantly when comparing the converted treatments with its reference (Table 1), allowing the effect of changes in managements to be examined without confounding effects related to soil texture. SSA, the amount of exchangeable Ca^2+^ and pH were significantly larger for AG than for A treatment.

**Table 1:** Soil characteristics. In case of statistical significance (*P* < 0.05) letters within rows denote significance for the comparison of G with GA and GBF, BF with BFG, and A with AG. For treatment abbreviations, see Fig. 1.

	G	GA	GBF		BF	BFG		A	AG
Texture^1^									
Clay < 2 μm	0.261	0.255	0.254		0.270	0.244		0.264	0.266
Silt 2–20 μm	0.272^b^	0.255^a^	0.256^a^		0.249	0.267		0.263	0.253
Silt 20–63 μm	0.319	0.335	0.337		0.335	0.338		0.318	0.332
Sand 63–2000 μm	0.148	0.155	0.153		0.146	0.151		0.155	0.149
Specific surface area (m^2^ g^−1^ minerals)^2^	78.4	77.4	75.8		59.1	63.3		67.9^a^	74.4^b^
Exchangeable cations and CEC									
Na^+^ (mmol_c_ kg^−1^ minerals)	0.7^b^	0.4^a^	0.5^a^		0.4	0.4		0.5^a^	0.6^b^
K^+^ (mmol_c_ kg^−1^ minerals)	5.8	6.9	5.4		3.3	4.5		6.3	5.8
Ca^2+^ (mmol_c_ kg^−1^ minerals)	144.4	134.2	142.4		95.0	88.7		102.5^a^	125.5^b^
Mg^2+^ (mmol_c_ kg^−1^ minerals)	4.6	3.9	4.4		5.4	4.8		4.0	3.9
Sum of bases (mmol_c_ kg^−1^ minerals)	155.6	145.3	152.6		104.1	98.5		113.3	135.8
CEC (mmol_c_ kg^−1^ minerals)	209.9	246.6	229.6		140.5	134.4		173.8	186.1
Base saturation (%)	74.4	60.8	67.2		75.5	73.1		65.5	74.6
pH (CaCl_2_)	5.4	5.2	5.4		5.9	5.6		5.1^a^	5.5^b^

1 kg kg^−1^ of mineral fraction and based on oven-dry weight.

2 Clay is included as a co-variable as it is significant and makes the treatment effect significant.

### 3.2 Soil organic matter fractions

**Fig. 2:**
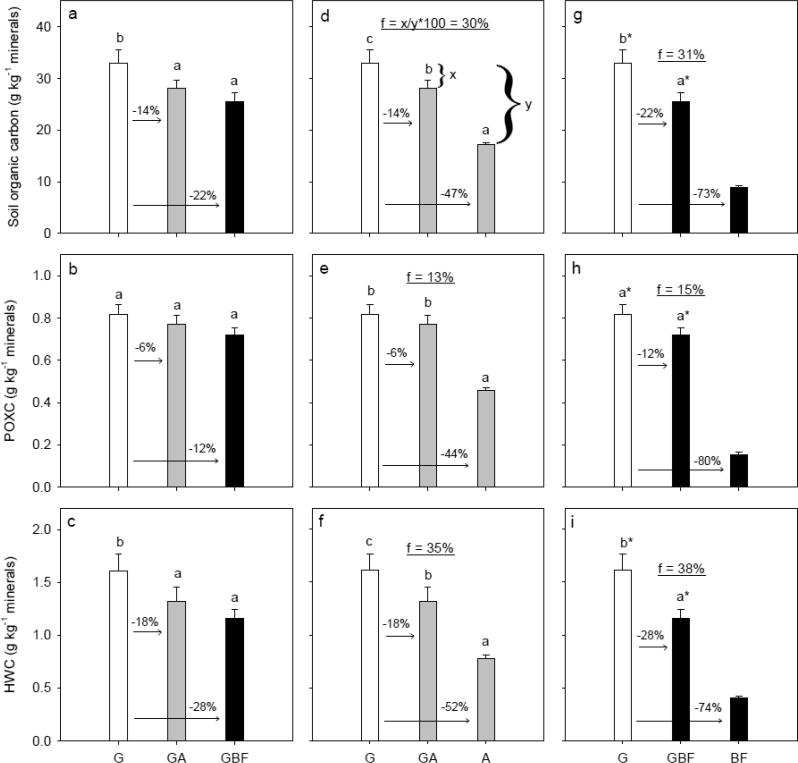
Degradation scenarios: Management system effects on soil organic carbon, permanganate oxidizable carbon (POXC), and hot water-extractable carbon (HWC). White, gray and black bar fills highlight treatments grass, arable and bare fallow, respectively, at time of sampling. Letters denote statistical significance at *P* < 0.05. An asterisk (*) indicates if BF is significantly different from GBF and G based on a pairwise *t*-test. The numbers above the arrows denote relative differences. The underlined number in the middle part of the figures denotes the decrease after six years in relation to the long-term decrease, and an example of the calculation is shown in Fig. d. For treatment abbreviations, see Fig. 1.

**Fig. 3:**
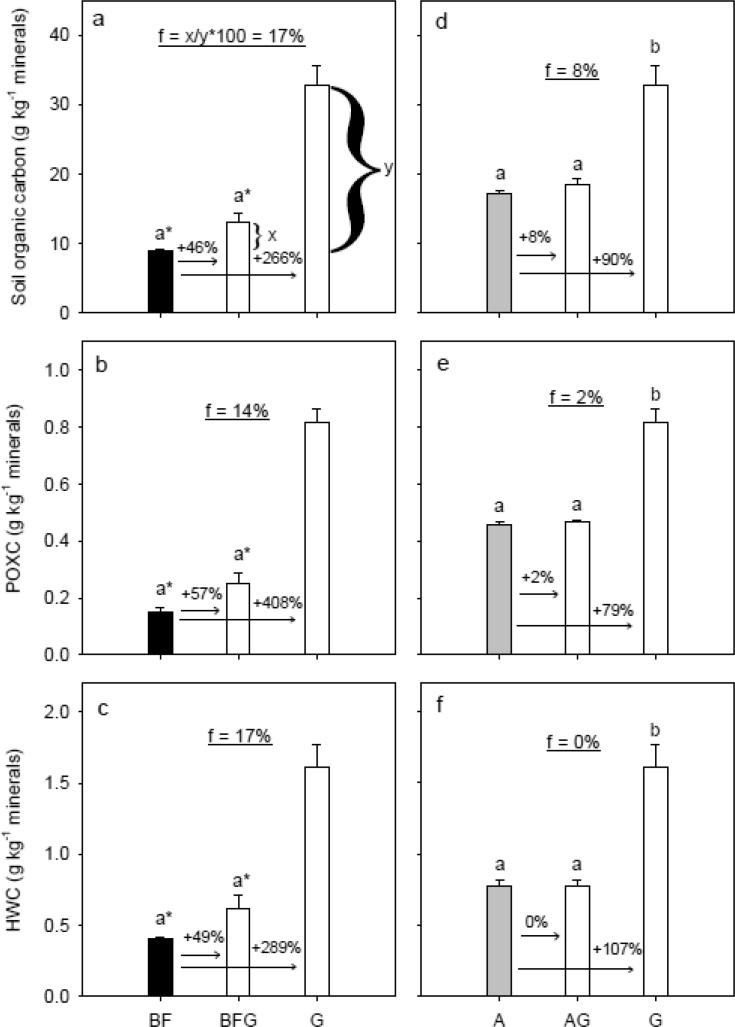
Restoration scenarios: Management system effects on soil organic carbon, permanganate oxidizable carbon (POXC), and hot water-extractable carbon (HWC). White, gray and black bar fills highlight treatments grass, arable and bare fallow, respectively, at time of sampling. Letters denote statistical significance at *P* < 0.05. An asterisk (*) indicates if G is significantly different from BF and BFG based on a pairwise *t*-test. The numbers above the arrows denote relative differences. The underlined number in the middle part of the figures denotes the increase after six years in relation to the long-term increase, and an example of the calculation is shown in Fig. a. For treatment abbreviations, see Fig. 1.

Concentrations of SOC and HWC were significantly lower for GBF and GA than for G (Fig. 2a and c). Similarly, POXC was lower for GBF and GA than for G, but not significantly and the decreases were less as compared to the changes in SOC and HWC (Fig. 2b). Concentrations of SOC, POXC and HWC were approx. 50% larger for BFG compared to BF, and marginally significant (SOC: *P* = 0.053, POXC: *P* = 0.055, HWC: *P* = 0.063), whereas the concentrations were not significantly different for AG compared to A (Fig. 3). POXC and HWC accounted for around 2.4% and 4.6% of total SOC, respectively. Correlations of POXC and HWC to SOC including data from all treatments at plot level can be seen in Fig. S1 in Supplementary material. Both POXC (Fig. S1a; broken stick regression, R^2^ = 0.96) and HWC (Fig. S1b; broken stick regression, R^2^ = 0.98) correlated well to SOC.

### 3.3 Soil structural stability and strength

**Fig. 4:**
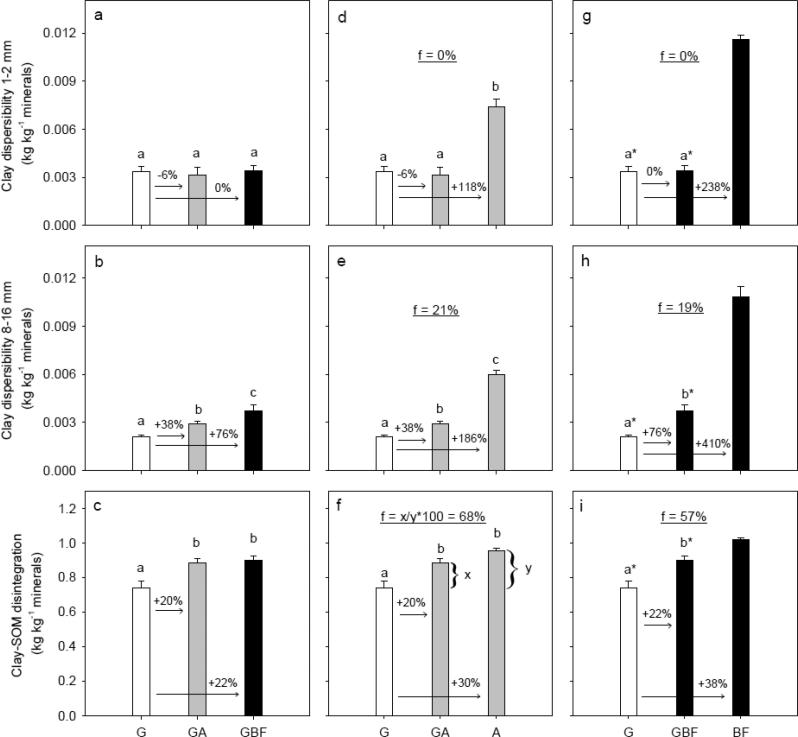
Degradation scenarios: Management system effects on clay dispersibility of 1–2 mm aggregates rewetted to −100 hPa, clay dispersibility of 8–16 mm aggregates rewetted to −100 hPa, and clay-SOM disintegration (the ratio between clay particles retrieved without SOM removal and with removal). White, gray and black bar fills highlight treatments grass, arable and bare fallow, respectively, at time of sampling. Letters denote statistical significance at *P* < 0.05. An asterisk (*) indicates if BF is significantly different from GBF and G based on a pairwise *t*-test. The numbers above the arrows denote relative differences. The underlined number in the middle part of the figures denotes the difference after six years in relation to the long-term difference, and an example of the calculation is shown in Fig. f. For treatment abbreviations, see Fig. 1.

There was no significant differences in the amount of dispersible clay of 1–2 mm aggregates (DispClay 1–2 mm) among G, GA and GBF, whereas DispClay 8–16 mm increased significantly in the order G < GA < GBF (Fig. 4a and b). Clay-SOM disintegration (DI) was significantly lower for the G treatment compared to GA and GBF (Fig. 4c). Tensile strength (*Y*) and Young’s modulus (*E*) of 8–16 mm aggregates did not differ significantly for G, GA and GBF, whereas rupture energy (*E*_sp_) was significantly lower for GA and GBF than for G (Table S1 in Supplementary material).

**Fig. 5:**
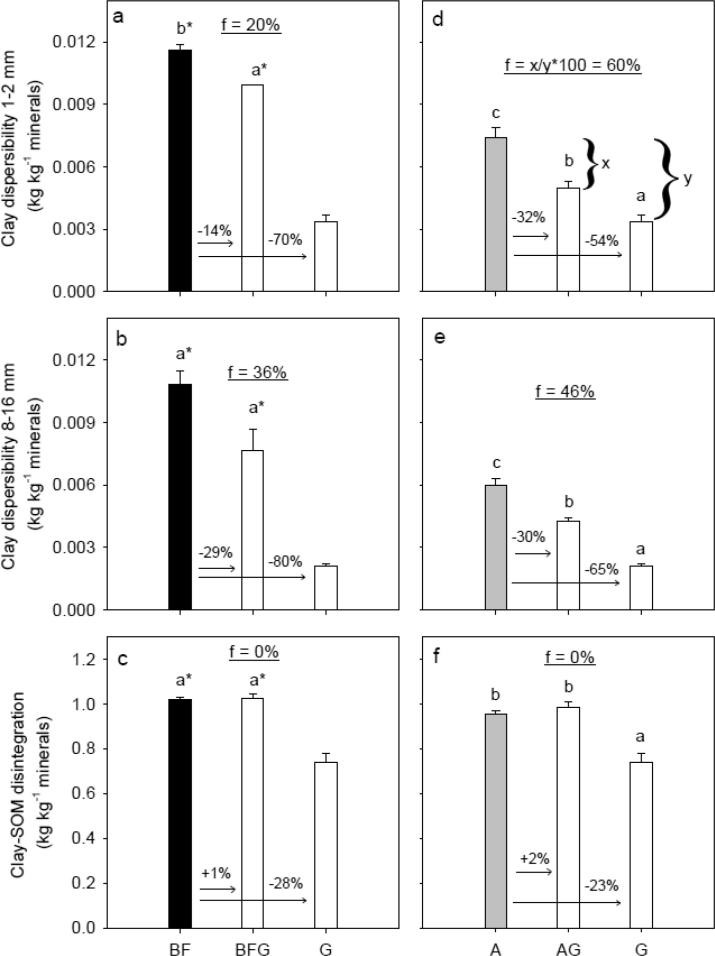
Restoration scenarios: Management system effects on clay dispersibility of 1–2 mm aggregates rewetted to −100 hPa, clay dispersibility of 8–16 mm aggregates rewetted to −100 hPa, and clay-SOM disintegration (the ratio between clay particles retrieved without SOM removal and with removal). White, gray and black bar fills highlight treatments grass, arable and bare fallow, respectively, at time of sampling. Letters denote statistical significance at *P* < 0.05. An asterisk (*) indicates if G is significantly different from BF and BFG based on a pairwise *t*-test. The numbers above the arrows denote relative differences. The underlined number in the middle part of the figures denotes the difference after six years in relation to the long-term difference, and an example of the calculation is shown in Fig. d. For treatment abbreviations, see Fig. 1.

DispClay 1–2 mm was significantly lower for BFG than for BF (Fig. 5a). A similar marginal significant lowering in DispClay 8–16 mm was seen (*P* = 0.072, Fig. 5b). DispClay 1–2 mm and DispClay 8–16 mm were significantly lower for AG than for A, and the relative reduction was approx. 30% (Fig. 5d and e). DI, *Y*, *E* and *E*_sp_ did not change significantly for BFG compared to BF and AG compared to A (Fig. 5c and f, and Table S1).

**Fig. 6:**
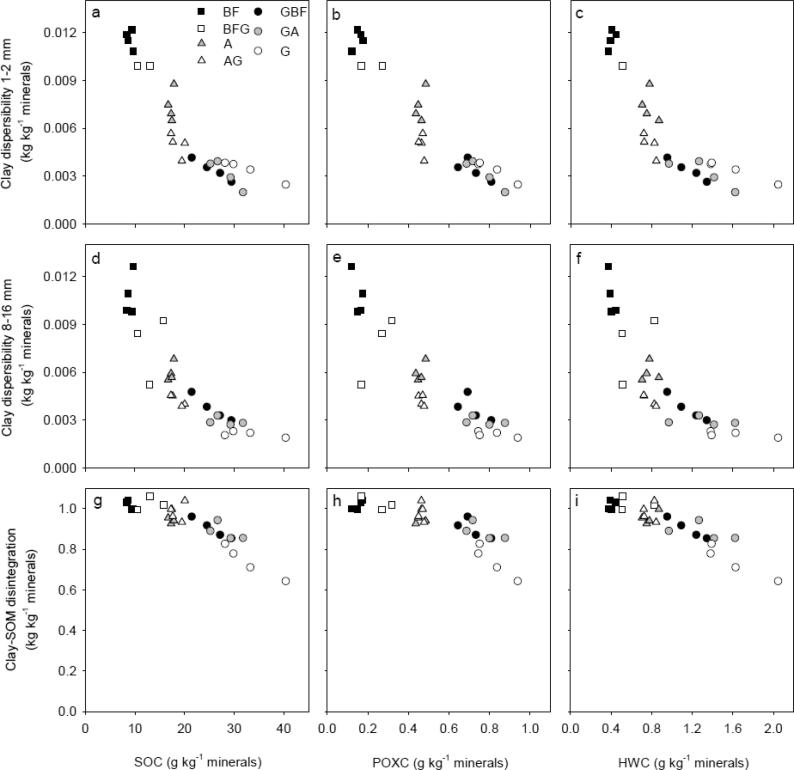
Soil structural stability measures plotted against soil organic carbon (SOC), permanganate oxidizable carbon (POXC) and hot water-extractable carbon (HWC) for the seven treatments at plot level. White, gray and black symbol fills highlight treatments grass, arable and bare fallow, respectively, at time of sampling. For treatment abbreviations, see Fig. 1.

**Fig. 7:**
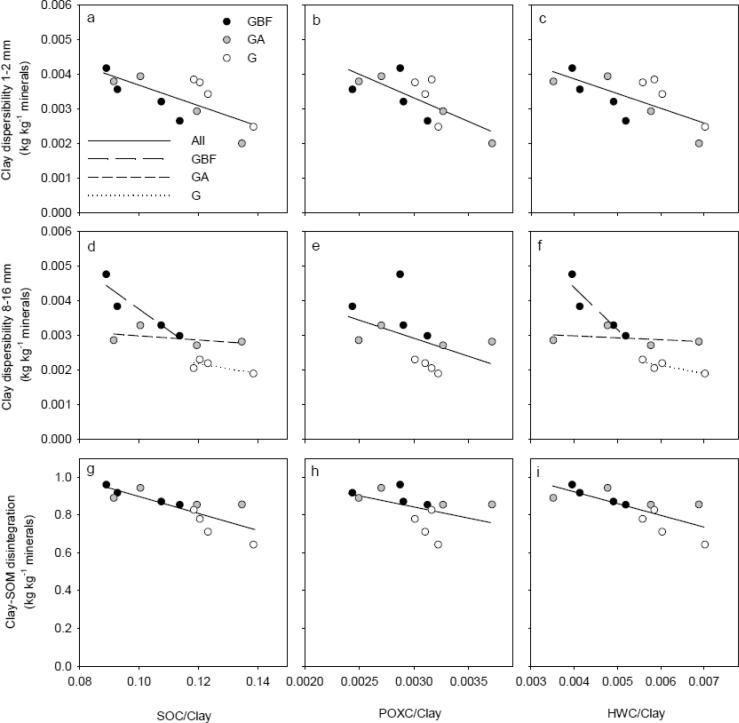
Correlations between soil structural stability measures and clay-content normalized expressions of soil organic carbon (SOC/Clay), permanganate oxidizable carbon (POXC/Clay) and hot water-extractable carbon (HWC/Clay) for GBF, GA and G at plot level. White, gray and black symbol fills highlight treatments grass, arable and bare fallow, respectively, at time of sampling. The linear regression including data for all treatments are indicated if the slope of linear regressions for individual treatments were not significantly different, whereas the linear regression for individual treatments are shown if the slope of the regressions were significantly different. See Table 2 for slopes, differences between slopes, and R^2^- and P-values. For treatment abbreviations, see Fig. 1.

Overall, soil structural stability increased with an increase in SOM fractions (Fig. 6). There was a small range in SOC, POXC and HWC within each treatment for BF and BFG and for A and AG, whereas the range in SOM fractions within G, GA and GBF were larger. Accordingly, linear models were employed to describe the correlations of SOC, POXC and HWC to DispClay 1–2 mm, DispClay 8–16 mm and DI for GBF, GA and G treatments only (Fig. 7). The SOM fractions were normalized to identical soil clay contents since this differed within treatments and is known to affect the SSS measures. For all three SSS measures, the coefficient of determination (R^2^) was highest when related to SOC/Clay (Table 2). However, there was a significant interaction between treatment and SOC/Clay as well as between treatment and HWC/Clay for DispClay 8–16 mm (Fig. 7d and f). Including the interaction term when describing the relation between SOC/Clay or HWC/Clay and DispClay 8–16 mm increased R^2^ from 0.60 to 0.89 and from 0.50 to 0.88, respectively (data not shown). Thus, the best model for describing DispClay 8–16 mm included the interaction term between treatment and SOC/Clay, explained 29%-units more of the variation than the model including SOC/Clay only, and had a lower AIC-value (8.4 vs 23.0). The relationship between SOC/Clay and DispClay 8–16 mm was not significant for G and GA, whereas it was almost significant for GBF (*P* = 0.071). In addition, the slope for GBF was significantly larger than for GA, and slightly larger than for G (*P* = 0.09). The slopes for G, GA and GBF when relating SOC/Clay to DispClay 1–2 mm and DI were not significantly different (no interaction).

**Table 2:** Slope, R^2^- and P-value for linear regressions of soil structural stability measures and clay-content normalized expressions of soil C and organic matter fractions individually for G, GA and GBF treatments as well as for all three treatments in combination. In case of statistical significance (*P* < 0.05) letters within rows denote significance for the comparison in slopes for G, GA and GBF. Clay dispersibility of 1–2 mm aggregates rewetted to −100 hPa (DispClay 1–2 mm), clay dispersibility of 8–16 mm aggregates rewetted to −100 hPa (DispClay 8–16 mm), clay-SOM disintegration (DI), soil organic carbon (SOC), permanganate oxidizable carbon (POXC), and hot water-extractable carbon (HWC). For treatment abbreviations, see Fig. 1.

Regression	G	GA	GBF	All
DispClay 1–2 mm vs SOC/Clay	−0.068	−0.044	−0.051	−0.030
R^2^- and P-value	0.99 (P = 0.005)	0.92 (P = 0.039)	0.90 (P = 0.051)	0.53 (P = 0.007)
DispClay 1–2 mm vs POXC/Clay	−4.660	−1.571	−1.063	−1.366
R^2^- and P-value	0.46 (P = 0.323)	0.94 (P = 0.029)	0.23 (P = 0.521)	0.52 (P = 0.008)
DispClay 1–2 mm vs HWC/Clay	−0.970	−0.564	−1.006	−0.426
R^2^- and P-value	0.95 (P = 0.027)	0.82 (P = 0.096)	0.89 (P = 0.058)	0.49 (P = 0.011)
DispClay 8–16 mm vs SOC/Clay	−0.014^ab^	−0.006^a^	−0.062^b^	−0.038
R^2^- and P-value	0.54 (P = 0.312)	0.22 (P = 0.536)	0.86 (P = 0.071)	0.60 (P = 0.003)
DispClay 8–16 mm vs POXC/Clay	−1.882	−0.224	−0.997	−1.045
R^2^- and P-value	0.96 (P = 0.021)	0.24 (P = 0.511)	0.14 (P = 0.632)	0.21 (P = 0.139)
DispClay 8–16 mm vs HWC/Clay	−0.242^ab^	−0.055^a^	−1.209^b^	−0.522
R^2^- and P-value	0.75 (P = 0.131)	0.10 (P = 0.687)	0.86 (P = 0.074)	0.50 (P = 0.010)
DI vs SOC/Clay	−8.037	−1.508	−3.972	−4.504
R^2^- and P-value	0.83 (P = 0.086)	0.48 (P = 0.310)	0.94 (P = 0.031)	0.64 (P = 0.001)
DI vs POXC/Clay	−406.73	−54.39	−81.03	−118.27
R^2^- and P-value	0.21 (P = 0.539)	0.51 (P = 0.288)	0.23 (P = 0.518)	0.20 (P = 0.143)
DI vs HWC/Clay	−109.39	−16.54	−78.24	−62.60
R^2^- and P-value	0.73 (P = 0.144)	0.31 (P = 0.439)	0.94 (P = 0.032)	0.55 (P = 0.006)

## 4 Discussion

### 4.1 Soil degradation after termination of grassland

Conversion of grassland to arable management (GA) introduces a change from a system with no tillage and permanent plant cover to a system with annual tillage and annual cereals, whereas the conversion of grassland to bare fallow (GBF) introduces a change to a system with intensive tillage and without plants. Consequently, the observed changes are a result of the combined effect of changed OM input and tillage. In this section, we address the effects of these soil degradation mechanisms in terms of changes in OM fractions (Fig. 2) and SSS (Fig. 4).

When grassland was terminated, the SOC content decreased by on average 14% and 22% for GA and GBF, respectively (Fig. 2a), due to reduced OM inputs and increased tillage intensity. Tillage is known to promote decomposition of SOM as it disrupts micro- and macro-aggregates, releasing entrapped OM, and increase soil aeration (Six et al., 1999). Besides the reduction in OM inputs, the quality of OM may also change in GA and GBF compared to G, and potentially contribute to the decline. The results accord with Attard et al. (2016), who found a rapid reduction in SOC after converting grassland to cropland. Changes in HWC (Fig. 2c) were only slightly higher than changes observed for SOC. This suggests that SOC and HWC show similar sensitivity to changes. This was also true for POXC although the differences were not statistically significant (Fig. 2b). The similar sensitivity to management changes for POXC, HWC and SOC found in this study contrasts with that of Bongiorno et al. (2019), who found that POXC was the most sensitive to changes in tillage and OM input in an analysis based on ten European long-term field experiments. Haynes and Swift (1990) found that the hot water-extractable carbohydrate-C was more sensitive to short-term changes in cropping histories than SOC suggesting that it is more relevant to focus on the carbohydrate-C in the hot water extract rather than C.

Changes in SSS due to changes in management depended on the size of macro-aggregates, supporting the theory that different stabilization mechanisms were important for stability of differently sized aggregates (Tisdall and Oades, 1982). The rapid increase in DispClay 8–16 mm and decrease in *E*_sp_ of similar-sized aggregates retrieved from GA and GBF may relate to destruction and loss of roots and fungal hyphae, these being important for stability at larger scale and both sensitive to tillage (Tisdall and Oades, 1982). Compared to G, DispClay 8–16 mm increased by an average of 38% and 76% for GA and GBF (Fig. 4b), respectively. This is a more dramatic change than the changes observed for SOC, HWC and POXC, indicating that the tillage-induced breakdown of binding agents may have overruled effects of OM fractions on the stability of larger aggregates. Similarly, Sparling et al., 1992, Grandy and Robertson, 2006 found that macro-aggregate (>2 mm) stability changed more rapidly than SOC content following conversion of permanent pasture to continuous maize cropping and tilling uncultivated soil, respectively. DispClay 8–16 mm was nearly constant across the four G and GA plots despite a range in the SOC/Clay ratio (Fig. 7d), indicating that management derived drivers such as root density were more important than SOC contents. In contrast, DispClay 8–16 mm increased with decreasing SOC for GBF. This may be due in part to additional tillage energy (Watts and Dexter, 1997) and to the loss of living roots and associated exudates under this management.

In contrast to DispClay 8–16 mm, DispClay 1–2 mm was similar for G, GA and GBF (Fig. 4a). This is surprising since the aggregate hierarchy concept (Oades and Waters, 1991) suggests similar response for >250 µm aggregates. The greater stability for smaller sized aggregates may relate to tillage-induced breakdown of larger sized aggregates in the former grassland soil followed by decomposition of OM released from aggregates as well as above- and belowground plant residues (Six et al., 1999). Formation of stable < 2 mm aggregates facilitated by microbial decomposition products may thus explain the delay in deterioration in DispClay 1–2 mm. Likewise, Sparling et al. (1992) found that >2 mm aggregates were more sensitive to grassland termination than 1–2 mm aggregates. DispClay 1–2 mm increased with decreasing SOC/Clay for both G, GA and GBF (Table 2) indicating that roots were less important for SSS in 1–2 mm aggregates than in 8–16 mm aggregates. Soil structural stability at microscale measured as DI increased with approx. 20% for both GA and GBF indicating the partial breakdown in GA and GBF of extremely stable organo-mineral associations that in treatment G ‘survived’ the extreme disturbance (Fig. 4c). SOC/Clay explained more of the variability in DI than both POXC/Clay and HWC/Clay (Table 2), which suggests that changes in DI were not strongly related to the supposed labile compounds. The comparable slopes for the relationship between SOC/Clay and DI for different treatments (Table 2) suggest that stability at microscale relates to SOC concentrations.

All structural stability measures correlated linearly to POXC/Clay irrespective of treatment (Fig. 7b, e and h). However, SOC/Clay as a sole predictor for all three SSS measures explained more of the variation than POXC/Clay and HWC/Clay as sole predictors. This is add odds with the conclusions of Bongiorno et al. (2019).

For DispClay 8–16 mm we found individual correlations to SOC/Clay and HWC/Clay within each management (Fig. 7d and f). This indicates that other drivers than SOC and HWC, respectively, are in play at this scale. Above, we hypothesize that the additional driver in play in our observations are roots and hyphae acting as stabilizing agents in 8–16 mm aggregates in the GA and G treatments. Our data thus point to the need for focusing on two mechanisms in SSS: 1) binding by roots and hyphae, and 2) bonding supported by microbial activity and residues (Oades, 1984).

### 4.2 Soil recovery by introduction of grassland

The conversion of bare fallow management to grassland (BFG) introduces a change from intensive tillage and no plant inputs to permanent plant cover and absence of tillage, while the conversion of arable management to grassland (AG) introduces a change from annual tillage and cereals to permanent plant cover and absence of tillage. In this section, we address the effects of these soil restoration mechanisms in terms of changes in OM fractions (Fig. 3) and SSS (Fig. 5).

Compared to the BF treatment, BFG shows similar relative increases in SOC, POXC and HWC (46–57%, Fig. 3a–c). For arable soil converted to grassland, SOC was slightly more responsive to changes in management than HWC and POXC (Fig. 3d–f). This suggests as for the degradation managements, that SOC, POXC and HWC show similar sensitivity to changes in restoration managements, which contrasts with Bongiorno et al. (2019).

DispClay 1–2 mm and DispClay 8–16 mm decreased by an average of 14% and 29% (Fig. 5a and b), respectively, when bare fallow was converted to grassland. However, small non-significant changes in SOC, POXC and HWC (Fig. 3d–f) had marked effects on both DispClay 1–2 mm and DispClay 8–16 mm when grassland replaced arable management (Fig. 5d and e). The more rapid change in macro-aggregate stability than in SOC content for AG agrees with results of Jastrow (1996) studying conversion of cultivated soil to tallgrass prairie. Poulton et al. (2018) also noted that small increases in SOC might have disproportionately large and beneficial effects on SSS.

Hirsch et al. (2017) found that microbial biomass and numbers of mesofauna increased when bare fallow and arable soils were converted to grassland. Further, the introduction of permanent grass increases root density (Attard et al., 2016) known to increase hyphal length (Schjønning et al., 2007). Roots and fungal hyphae may stabilize macro-aggregates (Tisdall and Oades, 1982), and microbial and faunal products derived from decomposition processes increase aggregate stability (Abiven et al., 2009). For both BFG and AG, the absence of tillage preserves the macro-aggregates and soil structure remains less disturbed, and the stabilizing agents are continuously replaced in soil under permanent grass. Thus, we suggest that the increase in macro-aggregate stability for BFG and AG may be due in part to the absence of tillage leading to development of the grass root system with associated positive effects on soil functions (Ajayi et al., 2019) including unrestricted aggregate formation and stabilization. The results from the restoration managements (Fig. 3, Fig. 5) thus align with results from the degradation managements (Fig. 2, Fig. 4) in pointing out the necessity to consider bonding as well as binding mechanisms in soil structural stabilization (Degens, 1997, Elmholt et al., 2008, Schjønning et al., 2007).

Although the importance of cations for SSS is considered minor in clayey soils (Bronick and Lal, 2005), the higher concentration of soluble Ca^2+^ in AG may potentially have contributed to the increased SSS by promoting flocculation of clay particles (Le Bissonnais, 1996).

### 4.3 Rate of change

Data from Hirsch et al., 2017, Rothamsted Research, 2018 show that the levels of SOM in the BF, A and G treatments had reached steady-state conditions when the Highfield-LUCE experiment was initiated. Therefore, changes in SOC and SSS six years after conversion can be related to equilibrium values for SOC and SSS, whereby the rate of change in the scenarios can be revealed (Fig. 2d–i, Fig. 3, Fig. 4d–i, and Fig. 5). The rate of change was calculated as f = x/y*100, where x and y denote the change in SOC and SSS after six years and at steady-state condition, respectively.

The change in SOC from grassland to bare fallow (GBF) and the reverse (BFG) correspond to 31% decrease and 17% increase of the range between the two reference treatments BF and G, respectively (Fig. 2g and Fig. 3a). The change from grassland to arable management (GA) and the reverse (AG) corresponds to 30% decrease and 8% increase of the range between A and G, respectively (Fig. 2d and Fig. 3d). These results agree with Johnston et al., 2009, Attard et al., 2016, who found that it was faster to lose than to restore SOC by management changes. The greater loss than gain in SOC could be due in part to differences in OM input in restoration and degradation managements. It may be difficult to establish grass in bare fallow and arable soil because of the poor structure, and Attard et al. (2016) found no change in SOC three years after cropland was converted to grassland, which was ascribed to the slow development of the root system. Nevertheless, SOC models such as RothC (Coleman and Jenkinson, 1996) and C-TOOL (Taghizadeh-Toosi et al., 2014) assume rate symmetry, i.e. equal change in both directions. Our findings challenge this assumption, and we encourage additional studies investigating rates of change in both directions.

DispClay 1–2 mm did not change for the grassland terminations. However, the introduction of grassland in bare fallow and arable soil correspond to 20% and 60% of the range between G, respectively (Fig. 5a and d). The stability of large macro-aggregates (DispClay 8–16 mm) was highly sensitive to management changes in both restoration and degradation scenarios. The decline in stability for grassland terminations correspond to ≈20% of the potential range (Fig. 4e and h), whereas the increase in stability in soil subjected to grassland corresponds to ≈40% of the range (Fig. 5b and e). Thus, with respect to SSS measures at macroscale, it was faster to restore SSS than to degrade SSS. Based on a compilation of four studies Kay (1990) showed differences in rates of change when planting forages on arable land. However, the studies only focused on restoration managements, and as for SOC knowledge on the rate of change in SSS in both directions are lacking. We encourage similar studies to examine if our findings are generally applicable.

Introduction of grassland did not affect DI at microscale after six years, whereas termination of grassland increased DI with ≈60% of the levels present in the corresponding reference treatments (Fig. 4f and i). To increase SSS at microscale, more than 2% SOC is needed for this soil (Fig. 6g and Fig. 4a in Jensen et al., 2019) regardless of soil management.

## 5 Conclusions

The Highfield-LUCE enabled us to quantify rates of change in OM fractions and soil structural stability (SSS) six years after the land use changed for soils subjected to contrasting long-term treatments. The loss of SOC in degradation scenarios was greater than the gain in SOC in the corresponding restoration scenarios. However, it was faster to gain SSS than to lose SSS at macro-aggregate scale. Accordingly, soil management affected SSS at macroscale beyond what is revealed from measuring changes in OM fractions. Based on our results, we suggest that the additional driver in play was binding agents. At microscale, SSS appeared to depend solely on the SOC content regardless of soil management. The results underline the need to include both bonding and binding mechanisms in the interpretation of changes in SSS induced by management.

## Declaration of Competing Interest

The authors declare that they have no known competing financial interests or personal relationships that could have appeared to influence the work reported in this paper.
