# Supplementary material for: Soil degradation and recovery – Changes in organic matter fractions and structural stability
Source: Geoderma. 2020 Apr 1;364:114181. doi: 10.1016/j.geoderma.2020.114181 (PMC7043339; doi:10.1016/j.geoderma.2020.114181)
Supplement: Supplementary data 1 [file mmc1.docx]

**Supplementary material for the article entitled: ”Soil degradation and recovery - changes in organic matter fractions and structural stability” by Jensen et al.**


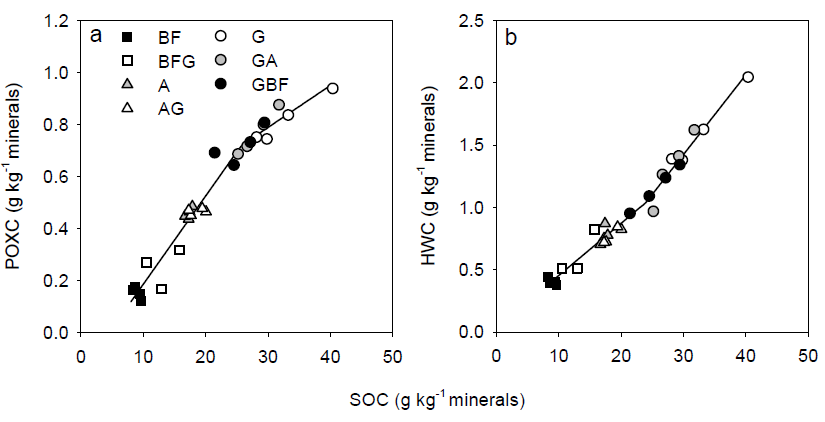


**Fig. S1.** (a) Permanganate oxidizable carbon (POXC) and (b) hot water-extractable carbon (HWC) as a function of soil organic carbon (SOC) for the seven treatments at plot level. White, gray and black symbol fills highlight treatments grass, arable and bare fallow, respectively, at time of sampling. The broken-stick models are indicated.

**Table S1.** Soil strength of 8-16 mm air-dry aggregates. In case of statistical significance (*P*<0.05) letters within rows denote significance for the comparison of G, GA and GBF, BF and BFG, and A and AG. For treatment abbreviations, see Fig. 1.

|  | G | GA | GBF |  | BF | BFG |  | A | AG |
| --- | --- | --- | --- | --- | --- | --- | --- | --- | --- |
| Tensile strength (*Y*, kPa) | 278 | 202 | 191 |  | 332 | 332 |  | 349 | 341 |
| Rupture energy (*E*_sp_, J kg^-1^) | 13.2^b^ | 5.5^a^ | 5.7^a^ |  | 9.4 | 8.0 |  | 10.8 | 10.7 |
| Young’s modulus (*E*, MPa) | 8.3 | 9.0 | 8.7 |  | 15.4 | 20.8 |  | 13.7 | 15.4 |
